# The perseverance time of informal carers for people with dementia: results of a two-year longitudinal follow-up study

**DOI:** 10.1186/s12912-015-0107-5

**Published:** 2015-11-06

**Authors:** Henk Kraijo, Job van Exel, Werner Brouwer

**Affiliations:** Julius Centre for Health Sciences and Primary Care, Utrecht MC, Utrecht, The Netherlands; Institute of Health Policy & Management, Erasmus University Rotterdam, Bayle Building, P.O. Box 1738, Rotterdam, 3000 DR The Netherlands

**Keywords:** Informal care, Burden, Perseverance time, Dementia, Longitudinal cohort study

## Abstract

**Background:**

Given the projected increase of people with dementia over the next few decades and the related demand for informal care, an important question for health policy makers is to what extent and for how long informal carers can be expected to provide care in a sustainable way. This study aimed to investigate the perseverance time of informal carers for people with dementia.

**Methods:**

A 2-year longitudinal cohort study was conducted. Questionnaires were used to collect data about the care situation, the impact of caregiving on carers and their need for support, and the anticipated and realized perseverance time of informal carers for people with dementia living at home. The data were analysed using bivariate and multivariate analyses.

**Results:**

Two hundred twenty-three carers for people with dementia were included in the study and 25 (11.2 %) dropped out during the follow-up. The results show that after 1 year, 74 (37.4 %) of 198 patients were still living at home, and after 2 years, 44 (22.2 %) patients were still living at home. The variables that were associated with this outcome were identified. When informal carers anticipated that their perseverance time would be less than 1 year, this was indicative of their actual perseverance time.

**Conclusions:**

Anticipated perseverance time provides a fair indication of the actual duration of informal care. It is most accurate when carers anticipate a limited rather than an unlimited perseverance time. Although further research is required to support these findings, the concept of perseverance time may be considered a useful additional instrument in health policy and clinical practice for monitoring carers’ need for support and for planning the transition of care from home to a nursing home.

## Background

Informal care is the care and support provided to patients by people from their social environment, normally in a non-professional and non-commercial capacity. The care can vary from temporary practical support to full-time care for an ill partner [1]. Informal care is an important part of the total care for many types of disease, especially when chronic illness is involved. One of the crucial questions regarding informal care is how long informal carers are able to provide informal care in a sustainable fashion. This question is becoming more important for health policy makers as trend studies suggest that the supply of informal care will decrease in coming decades, while at the same time the demand will grow [2–4]. Important reasons for the declining supply of informal care include: changes in the average size and composition of families, increasing (female) participation in the labour market, increasing geographical dispersion of families and increasing individualisation in society [5]. These social trends coincide with an expected increase in the demand for informal care due to an ageing population and an increased number of chronically ill patients. For example, in many countries the number of people with dementia is expected to double over the next few decades [6]. In addition, because of an anticipated future shortage of skilled care professionals and perpetually limited health care budgets, it seems unlikely that the formal health care sector will be able to fully accommodate this increase in demand. Hence, to limit the demand for formal care, governments seek to increase the involvement of informal carers and to prolong the duration of their involvement as much as possible.

The potential for doing this is, however, not unlimited, because the objective and subjective burdens of informal care can be substantial. Dementia is an illustrative example of an illness for which informal carers may experience substantial burdens, due not only to the prolonged and intense character of the informal care situation, but also to the progressive nature of the illness and the usually relatively advanced age of the carers. Many studies have shown that the impact of providing informal care to people with dementia can be profound [7–12]. Therefore, in order to know to what extent and for how long informal carers can be expected to provide care in a sustainable way, it is important for policy makers to understand what determines carers’ perseverance time [1] and how carers can be supported so that they can continue providing care. This will help decrease the demand for formal health care and delay nursing home admissions.

Increasing research attention is being devoted to informal care. For example, in order to identify factors which may help prolong the duration of caregiving, several studies have investigated the coping capacities and strategies of carers [13–15] and interventions aimed at reducing the burden and depression experienced by carers [16, 17]. An important concept in this area is carer resilience [18–21]. Several studies have shown that informal carers can be assisted in coping with the demands of informal care, and this lowers their perceived burden [9, 22, 23]. Other studies have examined the attitudes of informal carers regarding caregiving, respite care and institutionalisation [24–27]. All of this research has improved our understanding of the process of caregiving for relatives with dementia [28–30].

Another important branch of research has investigated the predictors of nursing home admission [31, 32]. Predicting admissions in individual cases remains difficult [33], while good timing of admissions is very important for several reasons. These include the general preference that both patients and carers have for caregiving at home as long as possible [34, 35] and the increasing scarcity of nursing home capacity [24].

Loeb [36] argued that an important step in addressing the perseverance of informal carers would be the development of an instrument capable of measuring anticipated perseverance time. Such a measure was recently proposed and validated [1]. In that study, the concept of perseverance time (Pt) was operationalized by asking informal carers for people with dementia the following question: “*If the informal care situation stays as it is now, how long will you be able to cope with the care*?” The study found that informal carers understood this line of questioning well, that convergent validity was moderate and content validity was fair to good, and concluded that Pt was useful for providing an indication of the time people expect to be able to continue care in light of the care situation and the burden it entails. An instrument capable of measuring perseverance time may help policy makers and practitioners support carers who expect a short perseverance time and can also help in planning timely admissions when unavoidable.

In the current paper, we report the results of a longitudinal study of the anticipated and realized perseverance time of informal carers for people with dementia. The sample of informal carers for people with dementia described in [1] was followed over a 2-year period. We registered Pt using the proposed instrument [1], along with a number of characteristics of the people with dementia, their informal carers and the caregiving situation. The aim of this longitudinal study was to investigate (i) how the care situations of people with dementia living at home developed over time, (ii) which caregiver, patient and care situation characteristics were associated with the patient living at home after one and two years, (iii) how the Pt of the informal carers developed over time, and (iv) whether Pt answers were indicative of the patient’s actual time of admission to a nursing home.

The structure of the rest of the paper is as follows. First, we describe the methods we used. Then we describe the results of our study. Finally, we discuss the results and present our conclusions.

## Methods

### Sample

This is a follow-up study of the cohort of carers for people with dementia described in [1]. The informal carers participating in this study were recruited between September 2007 and March 2008, in cooperation with the assessment agency of the Dutch Exceptional Medical Expenses Act in the Gooi and Vechtstreek region near Amsterdam, The Netherlands. While there is no formal registration for informal carers in The Netherlands, regional assessment agencies have registries of people diagnosed with dementia who are living at home and receiving formal help, such as home care. At our request, the assessment agency sent a letter to the home addresses of the 602 patients in their registry who were diagnosed with dementia, directed ‘To the primary informal carer of [patient name]’. The letter explained the purpose of our study and why the assessment agency had agreed to support the study by sending out this letter. In addition, the letter explained that the carer’s decision to participate in the study was voluntary and would not in any way affect formal care provision for their relative with dementia, that the data from the questionnaires that were returned were guaranteed to be anonymous (and how), and that we would assume that by returning the questionnaire they were granting us permission to use the data they provided for the purpose of this study (as described in the letter). A questionnaire and a stamped return envelope addressed to the assessment agency were attached to the letter. A reminder was sent four weeks later.

**Fig. 1:**
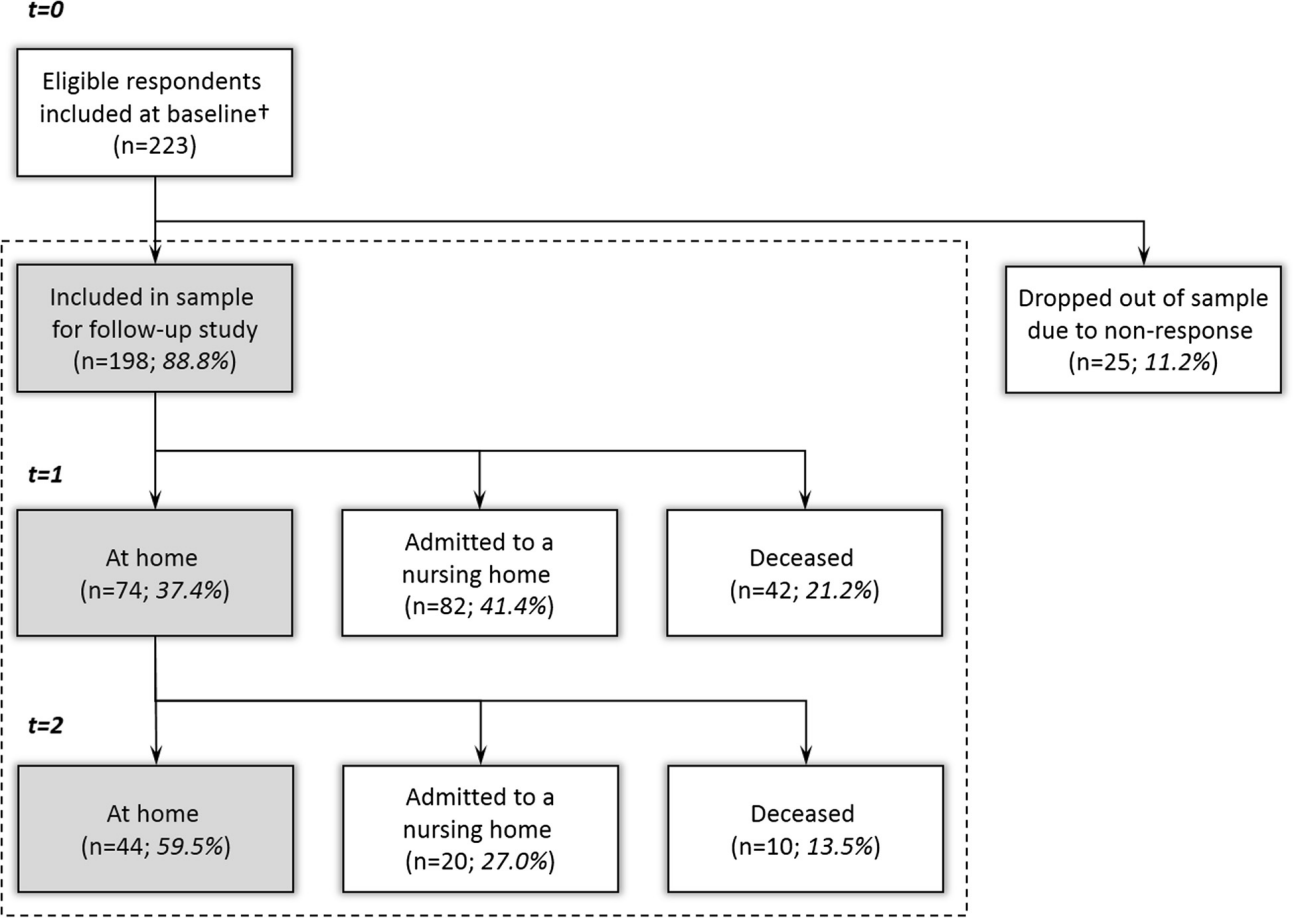
Development of care situations during the two-year follow-up. 198 of 223 eligible respondents were included in the study, of which 74 (37.4 %) still lived at home after one year and 44 (22.2 %) after two years. In total, 102 (51.5 %) participants were admitted to a nursing home during follow-up, and 52 (26.3 %) participants deceased

The investigators periodically received bundles of completed questionnaires from the assessment agency. Thus, the information available for this study consisted of the data provided by informal carers through our questionnaire, exclusive of information identifying respondents (i.e. their names and addresses) or patients (i.e. data from the assessment agency registry). A total of 292 envelopes were returned (gross response rate 48.5 %), of which 69 (23.6 %) had to be ignored because they contained (largely) incomplete questionnaires, or because respondents did not match the population of study. Consequently, completed baseline questionnaires were available from 223 informal carers (net response rate 37 %) [1]. These 223 carers were included in the analyses in this study (see Fig. 1).

The informal carers who agreed to participate in the study received a follow-up questionnaire 1 year (year 1) and—if applicable—2 years (year 2) after completing the first questionnaire (baseline). The data collection procedure at year 1 and year 2 was the same as described above for the baseline.

### Measures

The follow-up questionnaire administered at year 1 and year 2 was an abbreviated version of the baseline questionnaire used in the initial study [1], which consisted of a comprehensive set of questions about the informal carer, the dementia patient and the informal care situation (e.g., objective and subjective burden of care, need for support, adjustments in work and other activities). This baseline questionnaire, which was largely based on the iMTA valuation of informal care questionnaire (iVICQ) [37], is described in more detail in [1]. In the follow-up data collection at year 1 and year 2 we reduced the length of the baseline questionnaire in order to promote response. The follow-up questionnaire focussed on the key outcome measures for this longitudinal study, some of which are highlighted below.

The health status of the people with dementia and the carers was measured using a visual analogue scale (VAS) ranging between 0 (labelled ‘worst conceivable health’) and 10 (‘best conceivable health’). The patient’s care dependency was assessed using a VAS ranging between 0 (labelled ‘fully self-sufficient’) and 10 (‘fully care-dependent’).

The subjective burden of care was assessed with two short, validated instruments: the Caregiver Strain Index (CSI) [38] and the Self-Rated Burden (SRB) [39]. The CSI consists of 13 items describing problems which informal carers can experience. Respondents are asked to indicate whether they experience these problems (with no / yes answers), and the score is computed as a simple sum. The score ranges between 0, indicating no burden and 13, indicating that the carer experiences strain in all 13 problem areas. Substantial burden is defined as a CSI score of 7 or higher [38]. The SRB is a VAS on which informal carers are asked to indicate how burdensome they experience the informal care to be. The scale ranges between 0 (labelled ‘not at all straining’) and 10 (‘much too straining’) [39].

**Fig. 2:**
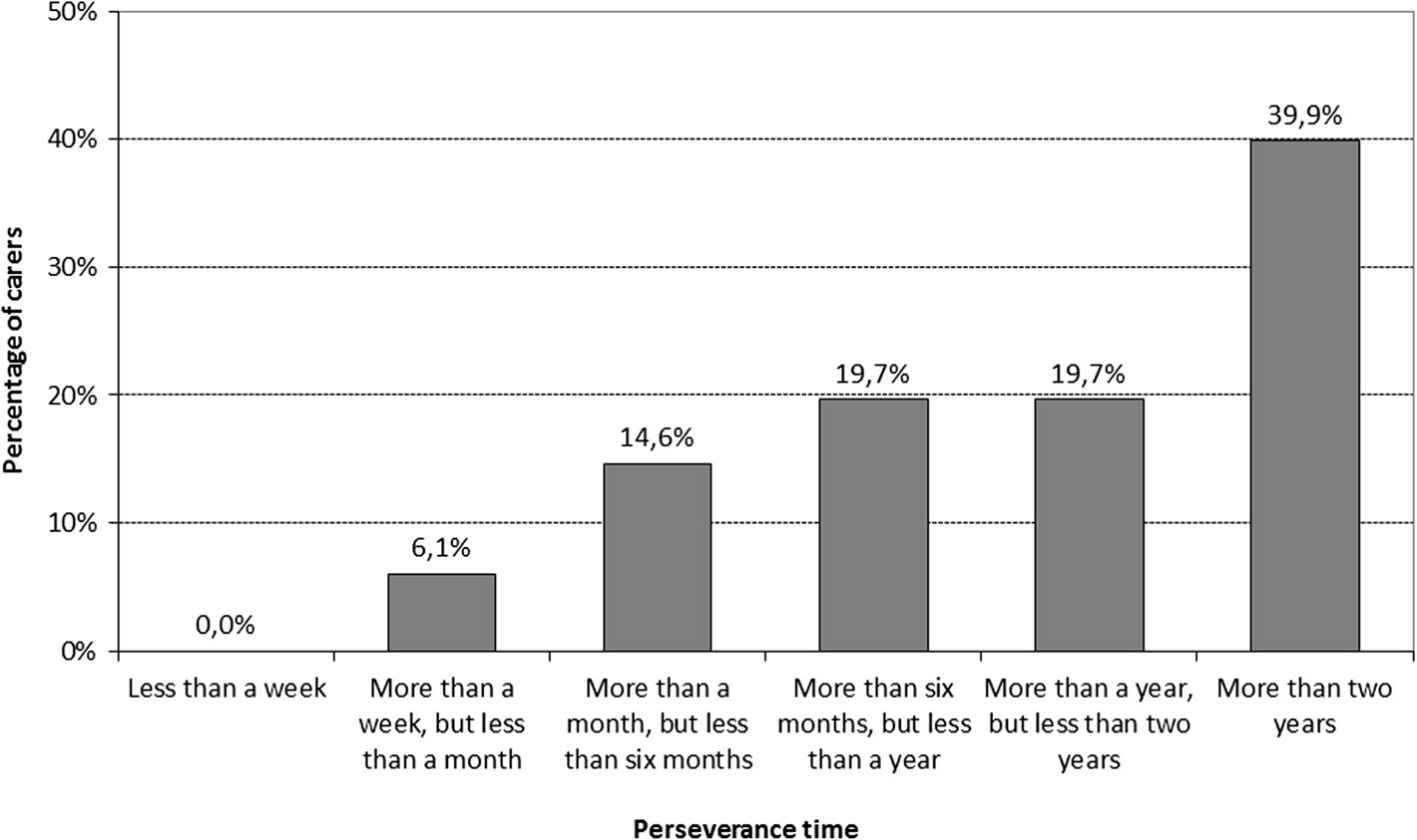
Anticipated perseverance time at baseline. About 20 % of carers expected to persevere in caregiving either less than six months, between six months and a year or between one and two years, while the largest group of about 40 % expected to persevere (at least) for the duration of the two-year follow-up period

Additionally, perseverance time (Pt) was assessed by asking informal carers: “If the informal care situation stays as it is now, how long will you be able to cope with the care?” The answer categories were: less than a week; more than a week, but less than a month; more than a month, but less than six months; more than six months, but less than one year; more than one year, but less than two years; more than two years [1] (see Fig. 2). The Pt (in months) was estimated by taking the middle value in a category (e.g. 9 months for the category ‘more than six months, but less than one year’). For the (open-ended) fourth category ‘more than two years’, the value was arbitrarily set at 30 months.

### Statistical analyses

Descriptive statistics of all variables are presented in mean and standard deviation for continuous variables and in numbers and percentages for categorical variables. Differences between sub-groups were tested using ANOVA in the case of continuous variables and chi-square in the case of categorical variables. Differences between two points in time (baseline, year 1, year 2) were tested using the paired sample *T*-Test. Logistic regressions were used for multivariate analyses. Statistical analyses were conducted with SPSS 20.0.

### Ethics approval

The study we present here was part of a larger project for which ethics approval was obtained from the Medical Ethics Committee (MEC) of Utrecht MC (protocol number 07-189/C; 26 July 2007). Although no additional approval was required for this specific study, the MEC verified the study protocol against the Dutch Data Protection Act and ruled that the study was designed carefully and that it met the codes of ‘good conduct’ and ‘good use’ of patient data. In conformity with these codes, informal carers provided informed consent by returning the questionnaire.

## Results

### Sample

As described in [1], 223 informal carers were included in this longitudinal study at baseline. During the two-year follow-up, 25 carers (11.2 %) dropped out by virtue of non-response (after reminders were sent). At baseline, this subgroup of carers reported a significantly higher burden of care than the carers that were retained for this study (mean (SD) CSI score 7.9 (3.0) vs. 6.5 (3.5), *p* < .05; SRB score 6.0 (2.2) vs. 4.8 (2.5), *p* < .05) and they were more often identified as being substantially strained (CSI score ≥ 7: *n* = 13 (52 %) vs. *n* = 62 (31 %), *p* < .05). Excluding these dropouts, the sample for the longitudinal analysis presented here consisted of 198 informal carers for people with dementia living at home at baseline (Fig. 1). At baseline, 79 (39.9 %) of the carers expected to persevere in caregiving (at least) for the duration of the two-year follow-up period (Fig. 2).

Table 1 presents descriptive statistics of the characteristics of the people with dementia, their carers and the caregiving situation.

**Table 1:** Sample and caregiving situation characteristics; mean (SD) or %

Characteristic		People with dementia living at home		
		Baseline (*n* = 198)	Year 1 (*n* = 74)	Year 2 (*n* = 44)
*Patients*				
Age (years)		81.3 (6.6)	81.8 (6.8)	82.1 (7.3)
Gender (female)		105 (53.0)	37 (50.0)	22 (50.0)
Marital status (single)		72 (36.4)	22 (29.7)	13 (29.5)
Lives alone (yes)		65 (32.8)	20 (27.0)	11 (25.0)
Health status (score 0–10)		5.8 (1.9)	5.0 (1.7)	5.5 (1.6)
Co-morbidity	- No	43 (21.7)	11 (14.9)	4 (9.1)
	- Mild	37 (18.7)	19 (25.7)	9 (20.5)
	- Moderate	79 (39.9)	34 (45.9)	19 (43.2)
	- Severe	39 (19.7)	10 (13.5)	12 (27.3)
Supervision	- Needs constant supervision	45 (22.7)	13 (17.6)	9 (20.5)
	- Can be left alone for one hour at the very most	46 (23.2)	29 (39.2)	12 (27.3)
	- Can easily be left alone for a couple of hours	107 (54.1)	32 (43.2)	23 (52.3)
Care dependence (score 0–10)		7.0 (2.4)	7.1 (2.0)	7.2 (2.0)
*Informal carers*				
Age (years)		66.6 (12.9)	69.6 (12.8)	70.3 (12.5)
- 65 years or older		112 (56.5)	48 (64.9)	28 (63.6)
Gender (female)		133 (67.2)	47 (63.5)	28 (63.6)
Marital status (single)		33 (16.7)	13 (17.6)	7 (15.9)
Children at home (yes)		19 (9.6)	7 (9.5)	4 (9.1)
Education level	- Low	24 (12.1)	8 (10.8)	5 (11.4)
	- Middle	120 (60.6)	46 (62.2)	24 (54.5)
	- High	54 (27.3)	20 (27)	15 (34.1)
Employed (yes)		57 (28.8)	16 (21.6)	8 (18.2)
Health status (score 0–10)		7.3 (1.6)	6.7 (1.8)	6.9 (1.5)
Well-being (score 0–10)		6.4 (1.8)	6.4 (2.0)	6.4 (1.8)
*Relationship*				
Care recipient is…	- Partner	109 (55.0)	48 (64.9)	29 (65.9)
	- Parent (in-law)	74 (37.4)	21 (28.4)	11 (25.0)
	- Other (family) relationship	15 (7.6)	5 (6.8)	4 (9.1)
Co-residents (yes)		116 (58.6)	50 (67.6)	31 (70.5)
*Objective burden*				
Duration of caregiving (years)		3.2 (2.2)	4.2 (2.5)	5.4 (2.8)
Intensity of caregiving	(days per week)	4.8 (2.8)	5.5 (2.5)	5.3 (2.6)
	(hours per week)	38.6 (41.5)	42.3 (42.9)	34.2 (34.4)
Formal care at home (yes)		146 (73.7)	68 (91.9)	41 (93.2)
- Hours per week, if yes		8.5 (13.2)	5.8 (6.6)	11.4 (21.6)
Formal day-care away from home (yes)		125 (63.1)	48 (64.9)	26 (59.1)
Support by other informal carers (yes)		108 (54.5)	33 (44.6)	20 (45.5)
- Hours per week, if yes		9.0 (11.2)	7.2 (5.6)	10.4 (9.8)
Adjustments:	- Adjusted working hours (if applicable^a^)	49 (24.6)	5 (6.3)	11 (25)
	- Reduced volunteer work (if applicable^b^)	81 (40.8)	15 (20.0)	4 (9.1)
	- Abandoned hobbies (if applicable^c^)	83 (41.7)	31 (41.9)	17 (38.6)
*Subjective burden*				
Caregiver Strain Index (score 0–13)		7.9 (3.0)	7.0 (3.5)	6.6 (2.7)
- Substantial strain (CSI score ≥ 7)		136 (68.7)	42 (56.8)	22 (50.0)
Self-Rated Burden (score 0–10)		6.0 (2.2)	5.5 (2.4)	5.6 (2.1)
Perseverance time (months)		17.8 (11.1)	20.0 (10.5)	22.1 (9.3)
*Desire for additional support*				
Help with caregiving activities (yes)		88 (44.4)	35 (47.3)	11 (25.0)
Emotional support (yes)		34 (17.2)	9 (12.2)	11 (25.0)
Respite (yes)		71 (35.9)	14 (18.9)	16 (36.4)
None (no)		30 (15.2)	26 (35.1)	17 (38.6)

^a^Number of respondents employed: *n* = 57 (baseline); *n* = 16 (year 1); *n* = 8 (year 2)

^b^Number of respondents doing volunteer work: *n* = 49 (baseline); *n* = 15 (year 1); *n* = 11 (year 2)

^c^Number of respondents with a hobby: *n* = 168 (baseline); *n* = 136 (year 1); *n* = 44 (year 2)

The average age of patients at baseline was 81.3 years (SD 6.6; range 60–97), and about half were women; these percentages do not change considerably when restricted to the patients retained during follow-up. 36 % of the patients were single and 33 % lived alone; these percentages decreased slightly among those who were still living at home at year 1 and at year 2. The average health of the patients was ranked at 5.8 (SD 1.9; range 0–10) and co-morbidities were reported for 78 % of the people with dementia; the percentage of patients without co-morbidities clearly declined over time in the patient group still living at home. Almost half of the patients needed (constant) supervision, and the average care dependence score was considerable, at 7.0 (SD 2.4; range 0–10).

The average age of the informal carers was 66.6 years (SD 12.9; range 35–93), over half were age 65 or more, and two out of three were women. A minority of the carers were single or had children under the age of 18 co-residing with them, 29 % were employed, and the percentage of carers with a job decreased considerably during the follow-up among carers for patients still living at home. The carers rated their health at an average of 7.3 (SD 1.6; range 0–10) and their happiness at an average of 6.4 (SD 1.8; range 1–9). More than half of the carers (55 %) were the dementia patient’s partner, and 59 % of the patients lived in the same house as the carer; both proportions clearly increased among the cases of patients continuing to live at home during the follow-up.

The carers reported that they had been providing informal care for the patient for 3.2 years on average (SD 2.2; range 0–7) and that their current task was intensive, taking up a substantial part of their time: 38.6 h a week on average (SD 41.5; range 1–126), spread over 4.8 days (SD 2.9; range 1–7). At baseline, 74 % of the patients received formal care at home, 63 % received day care away from home and about half received additional care from informal carers other than the primary carer (the respondent). During the follow-up, the proportion of patients living at home who received formal care increased, while the proportion receiving additional care from other informal carers decreased. A substantial proportion of carers reported that they had adjusted their working hours or social activities in order to persevere in the caregiving. For the majority of carers the burden of the caregiving situation was substantial, and only 15 % of the carers expressed no desire for additional support for their caregiving responsibilities. During the follow-up, the proportion of carers of patients living at home who reported substantial strain or a desire for support for their caregiving decreased considerably.

### Development of care situations

Figure 1 shows how the 198 care situations developed during the two-year follow-up. After one year, 74 of the people with dementia (37.4 %) still lived at home, and this number decreased to 44 patients (22.2 %) after two years. About half of the patients (*n* = 102; 51.5 %) were admitted to a nursing home during the follow-up period, the majority of these within one year of inclusion in this study, and about one quarter (*n* = 52; 26.3 %) had died. Here, it is relevant to note that the patients were on average diagnosed with dementia 3.2 years (SD 2.4; range 1–16) before inclusion in our sample and that the survival period after diagnosis usually varies between 3 and 9 years [40, 41].

### Variables associated with observed perseverance

Table 2 highlights the differences in the characteristics of the patients, carers and caregiving situation at baseline for the subsamples of patients still living at home, admitted to a nursing home and deceased at year 1.

**Table 2:** Differences in characteristics of the care situation at baseline between people with dementia living at home, admitted to a nursing home and deceased at year 1; mean (SD) or %

Characteristics		Baseline	Year 1			
		Total (*n* = 198)	Home (*n* = 74)	Nursing home (*n* = 82)	Deceased (*n* = 42)	p
*Patients*						
Age (years)		81.3 (6.6)	80.0 (6.8)	82.4 (6.0)	81.7 (7.0)	*
Gender (% female)		105 (53.0)	37 (50.0)	51 (62.2)	17 (40.5)	*
Marital status (% single)		72 (36.4)	22 (29.7)	36 (43.9)	14 (33.3)	n.s.
Lives alone (% yes)		65 (32.8)	20 (27.0)	31 (37.8)	14 (33.3)	n.s.
Health status (VAS 0–10)		5.8 (1.9)	5.9 (1.8)	6.1 (1.9)	5.3 (2.0)	*
Co-morbidity (% yes)		155 (78.3)	61 (82.4)	58 (70.7)	36 (85.7)	*
Needs constant supervision (% yes)		45 (22.7)	11 (14.9)	24 (29.3)	10 (23.8)	*
Care dependence (VAS 0–10)		7.0 (2.4)	6.5 (2.4)	7.4 (2.5)	7.2 (2.3)	**
*Informal carers*						
Age (years)		66.6 (12.9)	68.6 (12.8)	64.6 (13.0)	66.6 (12.7)	n.s.
Gender (% female)		133 (67.2)	47 (63.5)	53 (64.6)	33 (78.6)	n.s.
Marital status (% single)		33 (16.7)	13 (17.6)	16 (19.5)	4 (9.5)	n.s.
Children at home (% yes)		19 (9.6)	7 (9.5)	9 (11.0)	3 (7.1)	n.s.
Education level (% high)		54 (27.3)	20 (27.0)	23 (28.0)	11 (26.2)	n.s.
Employed (% yes)		57 (28.8)	16 (21.6)	31 (37.8)	10 (23.8)	*
Health status (VAS 0–10)		7.3 (1.6)	7.2 (1.8)	7.4 (1.4)	7.1 (1.6)	n.s.
Well-being (VAS 0–10)		6.4 (1.8)	6.5 (1.7)	6.6 (1.6)	5.8 (2.1)	*
*Relationship*						
Care recipient is partner (% yes)		109 (55.1)	48 (64.9)	36 (43.9)	25 (59.5)	**
Care recipient is parent (in-law) (% yes)		74 (37.4)	21 (28.4)	38 (46.3)	15 (35.7)	*
Co-residents (% yes)		116 (58.6)	50 (67.6)	41 (50.0)	25 (59.5)	*
*Objective burden*						
Duration of caregiving (years)		3.2 (2.0)	3.2 (2.1)	3.2 (1.8)	3.4 (1.8)	n.s.
Intensity of caregiving	(in days per week)	4.9 (2.7)	5.2 (2.6)	4.5 (2.7)	5.1 (2.7)	n.s.
	(in hours per week)	38.6 (41.5)	37.0 (42.2)	33.7 (47.7)	51.0 (36.7)	*
Formal care at home (% yes)		146 (73.7)	53 (71.6)	56 (68.3)	37 (88.1)	*
Support by other informal carers (% yes)		108 (54.5)	34 (45.9)	47 (57.3)	27 (64.3)	n.s.
Adjustments:	- Adjusted working hours (if applicable^a^)	49 (24.6)	0 (0.0)	24 (29.0)	21 (50.0)	**
	- Reduced volunteer work (if applicable^b^)	81 (40.8)	30 (40.0)	39 (47.8)	11 (27.3)	n.s.
	- Abandoned hobbies (if applicable^c^)	83 (41.7)	24 (32.3)	40 (48.6)	19 (44.1)	n.s.
*Subjective burden*						
Caregiver Strain Index		7.9 (3.0)	7.1 (3.0)	8.2 (2.7)	8.6 (2.8)	**
Self-Rated Burden		6.0 (2.2)	5.6 (2.3)	6.4 (1.9)	5.9 (2.2)	*
*Anticipated perseverance time*						
Anticipated perseverance time (in months)		17.9 (11.0)	22.2 (9.3)	14.8 (11.1)	16.4 (11.2)	***
% Pt > 2 years		79 (39.9)	40 (54.1)	24 (29.3)	15 (35.7)	***
% Pt > 1 year		118 (59.6)	58 (78.4)	39 (47.6)	21 (50.0)	***
% Pt > 6 months		157 (79.3)	70 (94.6)	55 (67.1)	32 (76.2)	***

^a^Number of respondents employed: *n* = 47 (total); *n* = 16 (home); *n* = 31 (nursing home)

^b^Number of respondents doing volunteer work: *n* = 38 (total); *n* = 15 (home); *n* = 23 (nursing home)

^c^Number of respondents with a hobby: *n* = 134 (total); *n* = 62 (home); *n* = 72 (nursing home)

*p < 0.10

**p < 0.05

***p < 0.01; n.s. = not significant

The patients still living at home after 1 year were on average younger and more were male. Fewer of those living at home needed constant supervision and they had on average a lower care dependence. Deceased patients had a lower health status and more comorbidities. Fewer carers of the patients still living at home were employed, while carers of deceased patients had on average a lower well-being. More patients still living at home were their carer’s partner, fewer were the carer’s parent (in-law), and more co-resided with the carer. The intensity of the care task for deceased patients was considerably greater, while these patients also more often received formal care at home. Fewer carers of patients still living at home had made adjustments to their work in order to provide care. On average, the subjective burden of care, measured with either CSI or SRB, was lower for the carers of patients who were still living at home, and their reported Pt was considerably higher. More than 3 out of 4 of the carers for patients still living at home at year 1 had anticipated being able to cope with the care task for at least a year at baseline, provided the informal care situation remained the same, while fewer than half of the carers for patients admitted to a nursing home had anticipated being able to cope.

Not many statistically significant differences were observed between the characteristics at year 1 of patients who were still living at home at year 2 (*n* = 44) and of those who had been admitted to a nursing home (*n* = 20) or had died (*n* = 10) by year 2. The main differences (not shown in table) concerned the subjective burden of the care situation: the mean (SD) CSI score was lower (6.2 (3.3) vs. 8.2 (3.4); *p* < .05) for carers of those who were still living at home at year 2, the mean (SD) SRB score was lower (4.8 (2.2) vs. 6.6 (2.3); *p* < .001), and the mean (SD) reported Pt was higher (24.3 (8.2) vs. 13.7 (10.4); *p* < .001). Moreover, 86.4 % of the carers for patients still living at home at year 2 had anticipated at year 1 being able to cope with the care task for at least another year (provided the informal care situation remained as it was at baseline), while this was true of only 40 % of the other carers (*p* < .001).

### Anticipated Pt

Figure 2 shows the carers’ anticipated Pt at baseline. A small proportion of the carers for people with dementia (6.1 %) anticipated they could only cope with the caregiving situation for less than one month, if it remained the same, while a large proportion (39.9 %) indicated that they could cope for at least the duration of the 2-year follow-up.

A very similar response pattern for the Pt question was observed at year 1 among the 74 carers of patients still living at home, namely: less than a week (0.0 %); more than a week, but less than a month (1.4 %); more than a month, but less than six months (12.2 %); more than six months, but less than 1 year (18.9 %); more than 1 year, but less than 2 years (20.3 %) and more than 2 years (47.3 %), respectively. A similar pattern also held at year 2 (*n* = 44): the percentages were 0.0, 0.0, 6.8, 13.6, 27.3 and 52.3 %, respectively.

### Anticipated Pt and observed perseverance

**Fig. 3:**
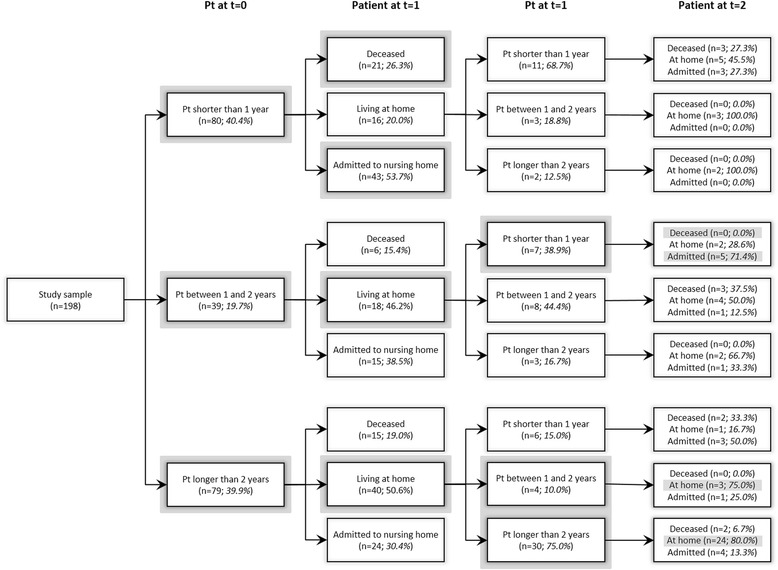
Anticipated and realized perseverance time. Comparison between anticipated and realized Pt of all carers in the sample throughout the follow-up study. The expected path based on anticipated Pt at baseline is shaded in grey

Table 3 compares the carers’ anticipated and realized Pt. When examining this comparison, it is important to bear in mind that while we asked for anticipated Pt based on the assumption that the care situation would not change, it often did change in reality. Still, Table 3 shows that a large proportion of the carers who anticipated at baseline that their Pt would be less than a year in fact anticipated this correctly (90.2 % of the 41 carers with Pt < 6 months; 69.2 % of the 39 carers with 6 months < Pt < 1 year; thus 80.0 % in aggregate). Similar proportions were observed at year 1 (i.e. 90.0 % and 64.3 %, respectively; 75.0 % in aggregate). The proportions at year 1 were lower when only patients admitted to a nursing home were considered (and deceased patients were excluded when calculating the percentage of carers whose anticipated Pt was realized). Fig. 3 provides full details of the comparison between anticipated and realized Pt.

**Table 3:** Anticipated and realized perseverance time during 2-year follow-up

	Baseline					Year 1		
	N	Patient home after 1 year (year 1)		Patient home after 2 years (year 2)		N	Patient home after 1 year (year 2)	
		*N* (% Pt cat.)	% realized Pt^a^	*N* (% Pt cat.)	% realized Pt^a^		*N* (% Pt cat.)	% realized Pt^a^
*Anticipated perseverance time*								
- less than 6 months	41	4 (9.8 %)	90.2 %/65.9 %	3 (7.3 %)	n.a.	10	1 (10.0 %)	90.0 %/80.0 %
- between 6 months and 1 year	39	12 (30.8 %)	69.2 %/41.0 %	5 (12.8 %)	n.a.	14	5 (35.7 %)	64.3 %/35.7 %
- between 1 year and 2 years	39	18 (46.2 %)	n.a.	8 (20.5 %)	79.5 %/51.3 %	15	10 (66.7 %)	n.a.
- more than 2 years	79	40 (50.6 %)	n.a.	28 (35.4 %)	35.4 %^b^	35	28 (80.0 %)	n.a.
*Perseverance time < 1 year*								
- yes	80	16 (20.0 %)	80.0 %/53.7 %	n.a.	n.a.	24	6 (25.0 %)	75.0 %/54.2 %
*Perseverance time < 2 years*								
- yes	119	n.a.	n.a.	16 (13.4 %)	86.6 %/58.8 %	n.a.	n.a.	n.a.

^a^Realized Pt including/excluding deceased patients

^b^Patient living at home

Carers who anticipated at baseline that their Pt would be between one and 2 years also largely anticipated this correctly, as 79.5 % of their patients no longer lived at home at year 2. Note, however, that a considerable number of these patients were admitted by year 1. A majority of carers who anticipated at baseline that their Pt would be at least 2 years did not realize the anticipated Pt, as only 35.4 % of their patients still lived at home at year 2. Overall, it appears that carers who indicated a limited Pt more often anticipated their Pt correctly, and particularly for shorter Pt intervals.

Table 4 shows associations between anticipated Pt > 1 year and characteristics of the people with dementia, their informal carers and the caregiving situation at baseline. Carers for female patients, who on average had slightly better health and lower care dependence, more frequently indicated an anticipated Pt of longer than 1 year than did carers for male patients, and so did carers who were younger rather than older, male rather than female, employed rather than unemployed, healthier rather than not so healthy, and happier rather than not. Fewer of the carers who were providing care to their partner rather than to a patient with a different relationship expected to cope for longer than 1 year, fewer who co-resided with the patient rather than living elsewhere expected to cope for longer than 1 year, and the same was true of carers with more rather than less intensive care tasks and carers who had abandoned rather than retained their hobbies in order to provide care. In general, carers experiencing higher strain from their care task indicated an anticipated Pt of less than 1 year more often than did other carers.

**Table 4:** Associations of anticipated Pt > 1 year at baseline and realized Pt > 1 year with characteristics of the care situation at baseline; mean (SD) or %

Characteristic		Anticipated Pt > 1 year			Realized Pt > 1 year		
		No (*n* = 80)	Yes (*n* = 118)	*p*	No (*n* = 60)	Yes (*n* = 58)	*p*
*Patients*							
Age (years)		82.0 (5.6)	80.9 (7.1)	n.s.	82.0 (7.2)	79.8 (6.0)	*
Gender (% female)		36 (45.0)	69 (58.5)	**	36 (60.0)	33 (56.9)	n.s.
Marital status (% single)		25 (31.3)	47 (39.8)	n.s.	27 (45.0)	20 (34.5)	n.s.
Lives alone (% yes)		23 (28.7)	42 (35.6)	n.s.	24 (40.0)	18 (31.0)	n.s.
Health status (VAS 0–10)		5.5 (2.0)	6.0 (1.8)	**	6.1 (1.9)	6.0 (1.7)	n.s.
Co-morbidity (% yes)		65 (81.2)	90 (76.3)	n.s.	42 (70.0)	48 (82.8)	*
Needs constant supervision (% yes)		22 (27.5)	23 (19.5)	n.s.	16 (26.7)	7 (12.1)	**
Care dependence (VAS 0–10)		7.5 (2.5)	6.7 (2.3)	**	7.2 (2.3)	6.2 (2.3)	**
*Informal carers*							
Age (years)		68.8 (12.0)	65.1 (13.3)	**	62.9 (13.4)	67.3 (13.0)	*
Gender (% female)		60 (75.0)	73 (61.9)	**	39 (65.0)	34 (58.6)	n.s.
Marital status (% single)		15 (18.8)	18 (15.3)	n.s.	7 (11.7)	11 (19.0)	n.s.
Children at home (% yes)		5 (6.2)	14 (11.9)	n.s.	7 (11.7)	7 (12.1)	n.s.
Education level (% high)		21 (26.3)	33 (28.0)	n.s.	17 (28.3)	16 (27.6)	n.s.
Employed (% yes)		18 (22.5)	39 (33.1)	*	25 (41.7)	14 (24.1)	**
Health status (VAS 0–10)		6.9 (1.7)	7.6 (1.4)	***	7.7 (1.5)	7.5 (1.4)	n.s.
Well-being (VAS 0–10)		5.9 (2.0)	6.7 (1.5)	***	6.7 (1.5)	6.7 (1.5)	n.s.
*Relationship*							
Care recipient is partner (% yes)		52 (65.0)	57 (48.3)	**	23 (38.3)	34 (58.6)	**
Care recipient is parent (in-law) (% yes)		23 (28.7)	51 (43.2)	**	31 (51.7)	20 (34.5)	**
Co-residents (% yes)		54 (67.5)	62 (52.5)	**	26 (43.3)	36 (62.1)	**
*Objective burden*							
Duration of caregiving (years)		3.4 (1.8)	3.1 (2.0)	n.s.	4.0 (2.0)	4.8 (2.1)	n.s.
Intensity of caregiving	(in days per week)	5.5 (2.4)	4.4 (2.8)	***	4.1 (2.8)	4.9 (2.8)	n.s.
	(in hours per week)	44.7 (40.8)	34.5 (41.7)	*	35.5 (43.6)	33.4 (40.0)	n.s.
Formal care at home (% yes)		59 (73.8)	87 (73.7)	n.s.	47 (78.3)	40 (69.0)	n.s.
Support by other informal carers (% yes)		44 (55.0)	64 (54.2)	n.s.	36 (60.0)	28 (48.3)	n.s.
Adjustments:	- Adjusted working hours (if applicable^a^)	27 (33.3)	24 (20.5)	n.s.	19 (32.0)	0 (0.0)	**
	- Reduced volunteer work (if applicable^b^)	31 (38.9)	49 (41.9)	n.s.	27 (44.4)	22 (38.5)	n.s.
	- Abandoned hobbies (if applicable^c^)	45 (56.5)	37 (31.3)	***	20 (33.3)	17 (29.2)	n.s.
*Subjective burden*							
Caregiver Strain Index		9.3 (2.6)	7.0 (2.9)	***	7.4 (2.6)	6.5 (3.1)	n.s.
Self-Rated Burden		7.4 (1.5)	5.0 (2.0)	***	5.1 (1.8)	4.9 (2.2)	n.s.

^a^Number of respondents employed: *n* = 18/39/14/25, respectively

^b^Number of respondents doing volunteer work: *n* = 18/31/13/18, respectively

^c^Number of respondents with a hobby: *n* = 69/99/48/51, respectively *p < 0.10

**p < 0.05

***p < 0.01; n.s. = not significant

Regarding the realization of an anticipated Pt > 1 year (*n* = 118) at baseline, carers more often anticipated their Pt correctly when they provided care to younger patients, patients with co-morbidities who did not need constant supervision, or patients who were not very care dependent. Carers who were older, those who were not employed, and those who provided care to their partner or co-resided with the patient also realized their anticipated Pt > 1 year more often. Finally, carers who had adjusted their working hours in order to persevere in their provision of care realized their anticipated Pt > 1 year less often than carers who had not adjusted their working hours.

Table 5 shows the results of logistic regression analyses with anticipated Pt > 1 year and realized Pt > 1 year as dependent variables and the characteristics listed in Table 4 as potential explanatory variables. Anticipated Pt > 1 year was negatively associated with patient being older, living alone and being partner of the carer, with abandoning hobbies and with subjective burden of care, and positively associated with reducing volunteer work. Realized Pt > 1 year was negatively associated with patient age and need for constant supervision, with subjective burden of care, and positively associated with patient having co-morbidity and carer age. In models excluding the subjective burden variable, the objective burden variable intensity of care giving reached statistical significance (i.e. days per week for anticipated Pt and hours per week for realized Pt). These multivariate associations are in line with the bivariate associations shown in Table 4, although fewer associations reach statistical significance.

**Table 5:** Logistic regression of anticipated Pt > 1 year at baseline and realized Pt > 1 year with characteristics of the care situation at baseline

Characteristic		Anticipated Pt > 1 year			Realized Pt > 1 year		
		B	S.E.	*p*	B	S.E.	*p*
*Patients*							
Age (years)		−0.09	0.04	.023	−0.08	0.04	.018
Gender (male)		0.13	0.49	n.s.	−0.02	0.61	n.s.
Lives alone (yes)		−1.47	0.65	.023			
Health status (VAS 0–10)		0.07	0.10	n.s.	−0.06	0.14	n.s.
Co-morbidity (yes)					1.28	0.60	.033
Needs constant supervision (yes)					−0.92	0.58	.113
*Informal carers*							
Age (years)		0.02	0.03	n.s.	0.05	0.02	.025
Gender (male)		0.34	0.49	n.s.	0.08	0.58	n.s.
Health status (VAS 0–10)		0.09	0.12	n.s.	−0.06	0.16	n.s.
*Relationship*							
Care recipient is partner (yes)		−1.88	1.04	.070			
*Objective burden*							
Adjustments:	- Reduced volunteer work (if applicable)	0.95	0.58	.102			
	- Abandoned hobbies (if applicable)	−0.71	0.38	.059			
*Subjective burden*							
Caregiver Strain Index		−0.33	0.08	.000	−0.26	0.10	.008
Constant		9.18	3.37	.006	5.58	3.91	.154
N			198			118	
Nagelkerke R^2^		.31			.29		
Percentage correct predicted		74.7			68.6		

In both regressions age, gender and health status of patient and carer were entered into the model, all other variables listed in Table 4 (except for self-rated measures of care dependence patient, burden and well-being carer) were added stepwise using the forward conditional function (p_in_ = .15 and p_out_ = .20 were used, considering the limited size of the sample and the explorative nature of the analysis)

*n.s.* not significant

## Discussion

This paper reported the findings of a longitudinal study of informal carers for patients with dementia who were living at home. The study focused on describing how the care situations developed over a two year follow-up period and the anticipated and observed perseverance times of the carers. In our study sample of 198 carers, only 74 (37.4 %) were still living at home after one year, and this number dropped to 44 (22.2 %) after two years. In the questionnaire, we directly asked the informal carers about their expected perseverance time (Pt), and we found that 80 % of the informal carers who anticipated a relatively short, limited Pt (i.e. less than one year) predicted their Pt correctly (or 53.7 % when deceased patients are excluded). The prediction was less accurate for carers who anticipated a longer or an unlimited Pt (i.e. more than two years).

Before highlighting some implications of our findings, we note a number of limitations of this study. First, we used a relatively small and specific sample consisting of 198 people registered as diagnosed with dementia and receiving formal care, from a single region in The Netherlands. No sample size calculation was conducted because the study was set-up to explore the newly developed measure of Pt; as this was the first study using this measure, its statistical properties were unknown. Therefore, all eligible people (*n* = 602) in the region of study were approached by mail with an invitation for their informal carer to participate in the baseline study [1]: 292 questionnaires were returned (gross response rate 49 %), of which 69 were inadmissible for various reasons, so that 223 informal carers could be included in the baseline study (net response rate 37 %); a total of 25 respondents dropped out during the follow up by virtue of non-response. Although we have no reason to believe that this selection of respondents –within a single region on the Netherlands- was problematic for the aims of this study, further investigation of the perseverance times of carers in larger samples and other regions –national as well as international- remains important. Second, the drop out during follow-up was selective. Specifically, particularly highly burdened carers dropped out, and this may have influenced our findings. It is crucial to investigate the perseverance time for this group, since it can be expected that the chance that patients cared for by this group would be admitted to nursing homes may be especially high. Third, we used only one patient group: patients with dementia. Given the nature of this disease, this led to a sample with certain characteristics (elderly patients, relatively old carers, deterioration of patients’ health, etc.). Given demographic and epidemiological projections, as well as the demand for the formal and informal care of people with dementia, knowledge regarding the carers for this patient group is extremely relevant. Still, it is also important to investigate perseverance time for carers of other groups of patients, since the results presented here may not be generalizable to other diseases. Fourth, the measure of Pt we used explicitly asks carers about anticipated perseverance time under the assumption that the caregiving situation remains ‘as it is now’. This was done to avoid the influence of (unrealistic) projections of the patient’s future health on the carers’ estimation of their perseverance time and to obtain an indication of the current severity of the caregiving burden. However, it must be emphasised that, for diseases like dementia, with its progressive nature, this assumption is very unlikely to hold true. Hence, the anticipated Pt may be an overestimation of the true perseverance time (e.g. if a patient deteriorates over time and the increased care demands are not fully met by others) or an underestimation (e.g. if support in caring from the patient’s social network or from professional carers increases more than the demand for care). One may expect that the carer’s anticipated Pt in contexts like dementia will most often be an overestimation of the actual perseverance time, given the deterioration of patients’ health over time.

Taking these limitations into consideration, we suggest that future studies should try to generate further insights about the effects of specific challenges or behaviours of care recipients (e.g., need for supervision) and changes in caregiving situations between measurements on the Pt of carers, investigate Pt at the time of the patient’s admission to a nursing home in relation to the main reason for admission, and add a follow-up question for the Pt categories in the questionnaire, asking carers to provide a more precise estimate of their perseverance time.

Notwithstanding the limitations of this study, our results have some important implications. First, informal carers who expect a relatively brief, limited perseverance time often predict this reasonably well. This means that if informal carers indicate that their perseverance time will be limited (i.e. less than a year), formal care can be anticipated, in the form of increasing formal support at home (to increase the carer’s perseverance time and the duration of the patient’s stay and care at home), facilitating timely admission into a nursing home (to prevent overstraining carers and causing crisis situations), or guaranteeing admission when the care situation at home is no longer sustainable (which reduces uncertainty [42] and thus supports carers in persevering as long as they can and wish to do so). In the case of carers anticipating a Pt of longer than one or two years, the accuracy of this estimation is lower. This may be because the caregiving situations did not remain stable and became more burdensome over time, thus reducing the carers’ Pt. Our results imply that while patients of carers who anticipate a Pt of less than a year are indeed at considerable risk of being admitted within that timeframe, patients of carers indicating a Pt of more than a year may still be admitted within a relatively short period. In sum, a short anticipated Pt appears to be a better predictor than a long one. Further investigation of why some carers overestimate their Pt (apart from deterioration of their patients’ health) remains important.

Previous longitudinal studies [43, 44] have documented how carers for people with dementia adapt to their role. In our study, as Fig. 3 also shows, a portion of the informal carers increased their estimated Pt over time; several indicated the same anticipated Pt at the different measurement moments. In some cases this may relate to an adaptation process on the part of informal carers which helps them to continue caring beyond points in time or levels of burden that they (or practitioners) had a priori expected to be possible [45, 46]. Measuring anticipated Pt may help to quantify and gain more insight into this adaptation process.

Moreover, more research could be focused on the tension between caregiving (for a relative with dementia) and participation in social activities and work, as well as on work productivity [33]. In this study, informal carers indicated that they had sacrificed unpaid or paid working hours and hobbies because of the informal caregiving situation. This may result in a prolonged stay at home for the patient. Policies facilitating such a trade-off, for instance through paid leaves of absence, may support informal carers in prolonging or intensifying their caring activities. Such policy options should be further explored [47].

Finally, perseverance time was recently introduced as a concept that complements the information from commonly used measures of care giving burden [1]. While such measures provide a good assessment of the status quo, Pt provides an indication of how long informal caregivers expect they can continue providing care, considering this status quo. Such information can be helpful to facilitate the optimal involvement of informal carers in the total care for people with dementia and the planning of a timely transition from care at home to nursing home care. This study underlines that the concept of Pt may indeed be a helpful additional measure for policy making and clinical practice. For example, carers expressing a limited perseverance time can be targeted with support that helps them prepare for the transition from care at home to nursing home care, or with extra or better, demand-oriented support at home so that transition can be postponed. The optimal policy is care situation specific, as carers’ motivations and preferences differ [24–27].

## Conclusions

Informal care is important in the context of diseases such as dementia. One of the key questions in this area, especially in light of the projected increase in the number of people with dementia and the related demand for informal care, is how to predict and influence informal carers’ perseverance time. Direct measurement of anticipated Pt has proved possible, and for carers who indicate a short, limited Pt this measure appears to have reasonable predictive accuracy. The concept of Pt may thus be a helpful policy tool for monitoring the need for support and planning the transition of care from home to nursing home. This should, however, be further investigated and confirmed in other samples and contexts. If confirmed, Pt may be a useful instrument in research on informal care and may directly facilitate health care policies and planning by allowing timely support of carers and facilitating timely admissions of patients to nursing homes.
